# A high-throughput energy-dispersive tender X-ray spectrometer for shot-to-shot sulfur measurements^1^

**DOI:** 10.1107/S1600577519002431

**Published:** 2019-04-02

**Authors:** Baxter Abraham, Stanislaw Nowak, Clemens Weninger, Rebecca Armenta, Jim Defever, David Day, Gabriella Carini, Kazutaka Nakahara, Alessandro Gallo, Silke Nelson, Dennis Nordlund, Thomas Kroll, Mark S. Hunter, Tim van Driel, Diling Zhu, Tsu-Chien Weng, Roberto Alonso-Mori, Dimosthenis Sokaras

**Affiliations:** a Linac Coherent Light Source, SLAC National Accelerator Laboratory, 2575 Sand Hill Road, Menlo Park, CA 94025, USA; b Stanford Synchrotron Radiation Lightsource, SLAC National Accelerator Laboratory, 2575 Sand Hill Road, Menlo Park, CA 94025, USA; c Brookhaven National Laboratory, Upton, NY 11973, USA; d SUNCAT Center for Interface Science and Catalysis, SLAC National Accelerator Laboratory, 2575 Sand Hill Road, Menlo Park, CA 94025, USA; e Department of Chemical Engineering, Stanford University, 443 Via Ortega, Stanford, CA 94305, USA

**Keywords:** X-ray emission spectroscopy, tender X-rays, X-ray absorption spectroscopy, X-ray free-electron lasers, photon-in photon-out spectroscopy methods

## Abstract

An X-ray emission spectrometer that can detect the sulfur *K*α emission lines with large throughput and a high energy resolution is presented. The instrument is based on a large *d*-spacing perfect Bragg analyzer that diffracts the sulfur *K*α emission at close to backscattering angles. This facilitates the application of efficient concepts routinely employed in hard X-ray spectrometers towards the tender X-ray regime. The instrument described in this work is based on an energy-dispersive von Hamos geometry that is well suited for photon-in photon-out spectroscopy at X-ray free-electron laser and synchrotron sources. Comparison of its performance with previously used instrumentation is presented through measurements using sulfur-containing species performed at the LCLS. It is shown that the overall signal intensity is increased by a factor of ∼15. Implementation of this approach in the design of a tender X-ray spectroscopy endstation for LCLS-II is also discussed.

## Abstract 2

An instrument for photon-in photon-out spectroscopy at XFELs in the energy range between soft and hard X-rays is presented.

## 1. Introduction   

The development of X-ray free-electron laser (XFEL) facilities such as FLASH, LCLS, SACLA, European XFEL, SwissFEL and PAL-XFEL has enabled the routine performance of a wide variety of X-ray experiments on ultrafast timescales (Spence, 2017 ▸; Bostedt *et al.*, 2016 ▸). Such studies span across a broad spectrum of physical and chemical phenomena in systems ranging from simple isolated atoms and small molecules to complex metalloproteins and nanomaterials (Shelby *et al.*, 2016 ▸; Fransson *et al.*, 2018 ▸; Tavella *et al.*, 2017 ▸). X-ray spectroscopy has been used in many of these XFEL studies to characterize ultrafast dynamics and capture intermediate transient states (Alonso-Mori & Yano, 2018 ▸). X-ray absorption (XAS) and X-ray emission spectroscopy (XES) are the most commonly employed photon-in photon-out spectroscopy methods. Although both techniques are able to provide complementary local electronic and atomic structural information with elemental specificity (Bergmann & Glatzel, 2009 ▸; Glatzel *et al.*, 2009 ▸), XES is generally more favorable for use in XFEL studies due to the stochastic nature of the self-amplified spontaneous emission (SASE) pulse spectrum (Alonso-Mori *et al.*, 2015 ▸). The utility of time-resolved XES has been extensively demonstrated for studying dynamics in photoactive molecules (Zhang *et al.*, 2014 ▸; Kjaer *et al.*, 2017 ▸), catalysts (Alonso-Mori *et al.*, 2016 ▸; Dell’Angela *et al.*, 2013 ▸) and metalloproteins (Mara *et al.*, 2017 ▸; Kern *et al.*, 2013 ▸). These studies have mainly focused on the soft (<1 keV) and hard (>5 keV) X-ray regimes due to the operational energy ranges available at existing XFEL endstations. However, the recent availability of Alvra at SwissFEL (Milne *et al.*, 2017 ▸) and the upcoming developments of LCLS-II (Schoenlein *et al.*, 2017 ▸) will provide systematic access to energies in the tender X-ray regime (∼1.5–5 keV). This will expand the possibilities for using X-ray spectroscopy to study important elements such as sulfur, phosphorus, chlorine, calcium, 4*d* transition metals and 5*f* actinides, enabling novel ultrafast studies on solar dyes, photocatalysts, biological systems, correlated materials and more (Chen *et al.*, 2014 ▸).

To facilitate the execution of time-resolved photon-in photon-out spectroscopy efficiently in the tender X-ray regime, the implementation of spectrometers with performance capabilities comparable with those used with hard X-rays is necessary. The measurement of shot-to-shot spectra is ideal to capture ultrafast dynamics, as it mitigates inconsistencies from XFEL pulse fluctuations (Yabashi *et al.*, 2006 ▸). Hard X-ray instruments for shot-to-shot experiments accomplish this by operating in an energy-dispersive geometry that allows an entire energy range to be simultaneously collected for every XFEL pulse. The dispersive von Hamos geometry is typically employed, using cylindrically bent Si or Ge analyzers to provide high resolution in the hard X-ray regime (Alonso-Mori *et al.*, 2012 ▸; Szlachetko *et al.*, 2012 ▸; Hoszowska & Dousse, 2004 ▸). Such instruments operate with an optimum throughput when the energies of interest are diffracted close to backscattering angles (∼70–89°), which maximizes the detection angle per unit energy. Given the numerous accessible Si/Ge crystal cuts, practically all hard X-ray energies can be analyzed at close to backscattering angles by selecting a Si/Ge cut with a proper *d*-spacing (Sokaras *et al.*, 2013 ▸).

When moving to the tender X-ray regime, this flexibility is no longer readily available. The commonly available materials (*e.g.* Si, Ge, SiO_2_) that can be machined and bent in typical curved geometries do not provide the fine variability in their *d*-spacing required to select energies within the tender range for diffraction at close to backscattering angles. This limitation has led to the development of energy-dispersive tender X-ray spectrometers that operate over wider angular ranges (down to ∼30°) using common analyzers such as Si(111), Si(220), InSb(110) and various cuts of SiO_2_ or less conventional LiF(200), PET(002), EDDT(0202) and TIAP(001) crystals for lower energies. These dispersive instruments use either a von Hamos approach or an inside-Rowland circle geometry with Johann or Johansson bent optics (Stojanoff *et al.*, 1992 ▸; Hoszowska *et al.*, 1996 ▸; Welter *et al.*, 2005 ▸; Hudson *et al.*, 2007 ▸; Journel *et al.*, 2009 ▸; Kavčič *et al.*, 2012 ▸; Rehanek *et al.*, 2018 ▸; Nowak *et al.*, 2019 ▸). Although these approaches have successfully enabled tender X-ray emission spectroscopy in synchrotron radiation facilities (Thomas *et al.*, 2015 ▸; Mori *et al.*, 2010 ▸; Alonso-Mori *et al.*, 2009 ▸; Butorin *et al.*, 2018 ▸), they have efficiency limitations when operating away from backscattering. This is due to the steep dependence of both the solid angle of detection and the beam size contribution to the energy resolution with the diffraction angle (Bergmann & Cramer, 1998 ▸; Sun *et al.*, 2015 ▸; Glatzel *et al.*, 2016 ▸). The limitation on throughput can be problematic for weak shot-to-shot XFEL measurements, where the signal from a single shot needs to be detected with an adequate signal-to-background ratio (Alonso-Mori *et al.*, 2015 ▸).

Here, we present a von Hamos tender X-ray spectrometer using a large *d*-spacing crystal analyzer that diffracts sulfur *K*α emission lines close to backscattering angles. This development allows the transfer of some of the advantages of well established hard X-ray instruments to the tender X-ray regime, leading to high throughput and high-resolution detection. Within the manuscript we illustrate the capabilities of the instrument through measurements on the sulfur *K*α emission from several species at LCLS. The design of a dedicated instrument for LCLS-II based on this progress is also discussed below.

## 2. Instrument overview   

**Figure 1:**
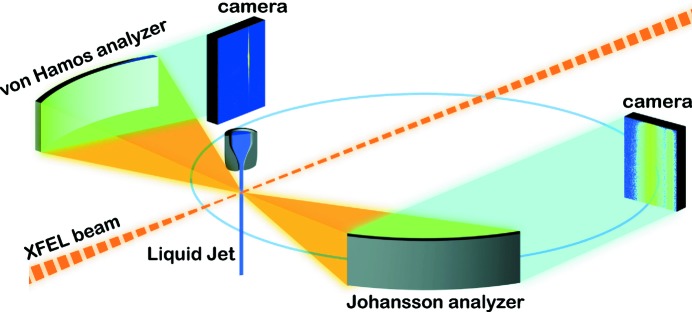
LiNbO_3_ 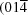) von Hamos and Si(111) Johansson spectrometers acquired experimental data simultaneously. The dual instrument experimental scheme is depicted. X-ray emission induced by LCLS pulses incident on the flowing sample is collected by both crystal analyzers and recorded shot-to-shot by position-sensitive detectors. The off-Rowland circle sample leads to dispersion in the Johansson geometry, whereas the von Hamos dispersion provides focusing of spectra to a line.

The spectrometer presented here, adapted to the tender X-ray regime, builds on the design of the hard X-ray von Hamos instrument presently based at LCLS (Alonso-Mori *et al.*, 2012 ▸). The dispersive von Hamos geometry is depicted in Fig. 1 ▸. A cylindrically bent perfect Bragg crystal analyzer is placed perpendicularly to the incident X-ray beam, which diffracts X-rays emitted from the sample and focuses them to a line on the detector plane. The analyzer is positioned above the horizontal plane of the sample according to the required Bragg angle. Energy dispersion occurs vertically from the crystal analyzer onto a 2D camera detector that lies directly above the sample. The signal is integrated over the horizontal axis of the detector to produce a spectrum. The vertical size of the crystal analyzer defines the accepted Bragg angles and thus the diffracted energy range, while the curvature provides horizontal focusing. The analyzer is mounted on a motorized platform with three degrees of freedom for alignment. Tilt around the axis defined by the curved direction of the crystal can be controlled to adjust the Bragg angle, rotation around the flat vertical axis of the crystal defines the position of the signal laterally on the detector and forward–backward radial motion allows adjustment of the focus. The spectrometer features a single 100 mm (horizontal) × 25 mm (vertical) lithium niobate crystal [LiNbO_3_ 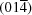], cylindrically bent to a 500 mm radius of curvature. The entire configuration is enclosed in a helium-purged chamber.

The use of a LiNbO_3_ 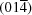 crystal analyzer is the key component for the efficient collection. The relatively large *d*-spacing of this material (2*d* ≃ 5.47 Å) leads to diffraction of the sulfur *K*α emission at a Bragg angle of ∼78.8°. This near-backscattering geometry allows for X-ray emission to be collected with significantly enhanced sensitivity in comparison with existing instruments configured with Bragg angles below 60° (see below). When operated at 78.8°, an energy range of 22 eV is dispersed by the analyzer and concurrently read out by the position-sensitive detector. Since the crystal analyzer covers a total solid angle of 9.8 millisterad, this provides a solid angle per energy of 0.45 millisterad eV^−1^. The position-sensitive ePix100 detector (Carini *et al.*, 2016 ▸) contains a 704 × 768 array of 50 µm^2^ pixels with a readout rate of 120 Hz, matching the LCLS repetition rate.

The achievable energy resolution of the instrument is constrained by geometrical factors from the incident beam size and detector pixel size (Δ*E*_geom_), as well as the Darwin width of the crystal (Δ*E*_Darwin_ = 0.2 eV) (Gog *et al.*, 2013 ▸). Imperfections in the crystal lattice (Δ*E*_imperf_ ≃ 0.2 eV) attributable to stress-induced distortion of the lattice planes and/or mosaicity further contribute to broadening. Under the conditions described, and including the vertical beam size of 25 µm and 50 µm^2^ detector pixels, the geometrical contribution is calculated to be 0.07 eV (Alonso-Mori *et al.*, 2012 ▸). The lower bound on the overall resolution is thus estimated at Δ*E*_total_ = 0.3 eV, resulting in a resolving power of ∼8000 (*E*/Δ*E*), by propagating these contributions according to
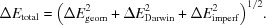
In this instrumental configuration, complete spectra can therefore be collected with high throughput and resolution at tender X-ray energies on a shot-to-shot basis. Multiple photon-in photon-out methods are compatible with the spectrometer design and can be performed with high efficiency in the tender X-ray regime, including XES, RXES (resonant XES) and HERFD-XAS (high-energy resolution fluorescence detected XAS). As such, this design is to be implemented in an upgraded instrument specialized for use at LCLS-II. An additional order of magnitude improvement to the collection efficiency will be achieved by increasing the solid angle of detection through the incorporation of additional crystal analyzers (4×) with reduced focal length (250 mm). Alternative crystal cuts with a variety of *d*-spacings will also be employed in order to fulfill the Bragg condition at close to backscattering angles for energies across the entire tender X-ray regime.

## 3. Results   

Measurements at the sulfur *K*α are presented for several compounds. Firstly, results from ammonium sulfate, (NH_4_)_2_SO_4_, are compared using the von Hamos spectrometer and a previously developed Johansson spectrometer described by Nowak *et al.* (2019 ▸). In brief, the Johansson spectrometer is set on a 0.5 m Rowland circle radius and detects the sulfur *K*α characteristic line at a 58.9° Bragg angle by using a Si(111) Johansson analyzer [700 mm (horizontal) × 15 mm (vertical)], featuring a resolving power of ∼6600 (*E*/Δ*E*). The sample was positioned 200 mm inside the Rowland circle of the Johansson analyzer. In this configuration, the spectrometer captures an energy range of ∼60 eV. An ePix100 camera, the same model used for the von Hamos spectrometer, is used as a position-sensitive detector.

XES data were collected simultaneously with both instruments as illustrated in Fig. 1 ▸. This dual instrument configuration was commissioned at beamline 6-2 of SSRL and experiments were performed at the XPP instrument of the LCLS (Chollet *et al.*, 2015 ▸). The samples were delivered through a cylindrical continuous-flow liquid jet of 50 µm thickness at a flow rate of 1 ml min^−1^. X-ray pulses were delivered at 120 Hz with a pulse length of 50 fs. The incident LCLS pink beam was centered at 6 keV, well above the sulfur *K*-edge, in order to maximize beamline transmission, albeit at the expense of a reduced absorption cross section. The incident X-ray spot size was 25 µm on the sample, resulting in a fluence of 400 J cm^−2^. The von Hamos instrument was contained in plexiglass and kept under a helium atmosphere, while the Johansson instrument was kept under vacuum at 10^−6^ mbar with an 8 µm polyimide window to separate the vacuum and He atmosphere of the sample environment. The emission signal was integrated over a period of ∼10 min.

**Figure 2:**
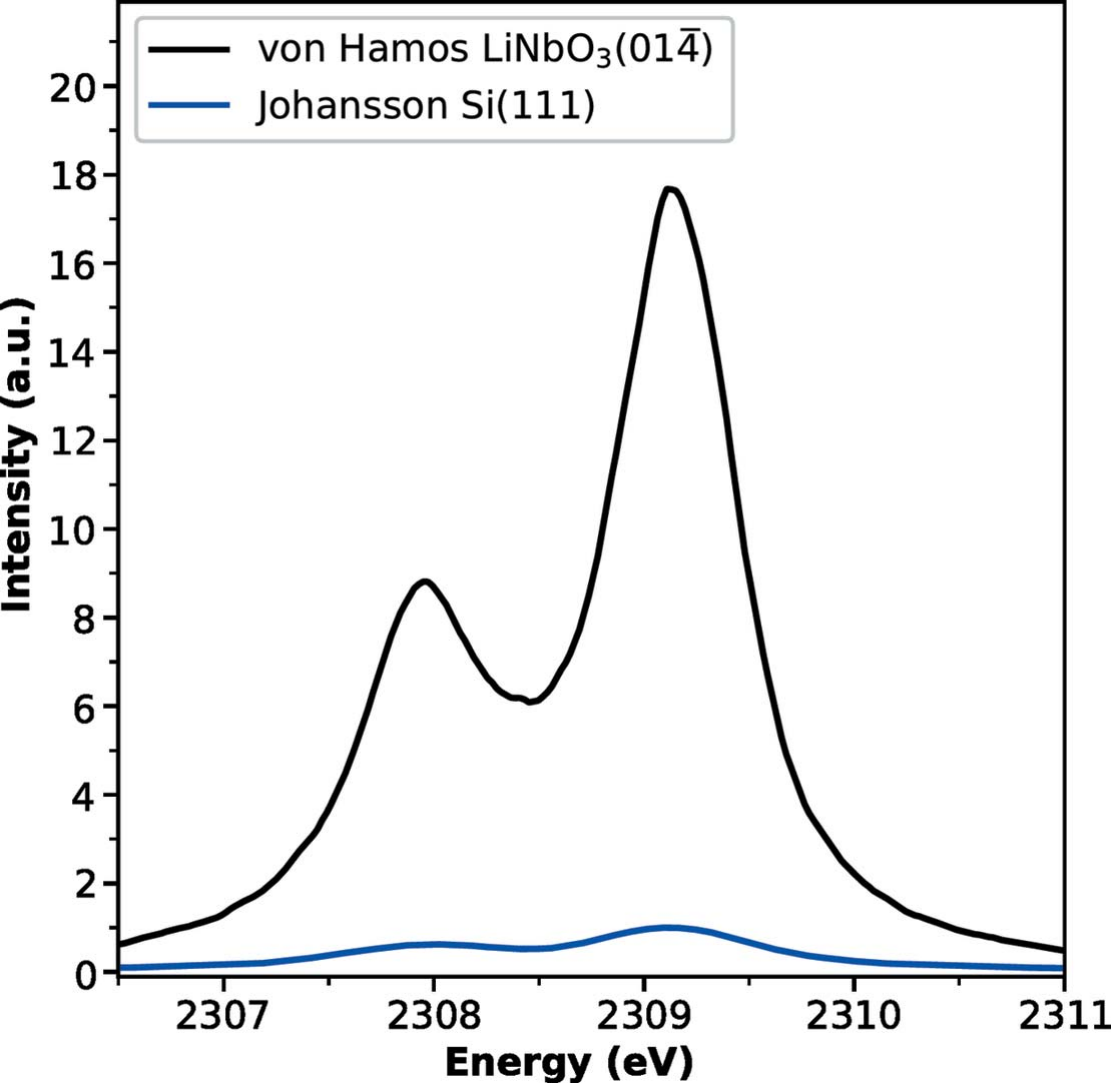
Comparison of the sulfur *K*α XES from (NH_4_)_2_SO_4_ using different detection schemes. The intensity of the signal measured by the lithium niobate von Hamos analyzer is ∼15 times greater than by the silicon-based Johansson analyzer.

The resulting *K*α spectra are displayed in Fig. 2 ▸ for comparison. Both instruments were able to accurately capture the full *K*α spectrum in a dispersive shot-to-shot operation. Notably, the overall signal intensity collected by the LiNbO_3_ von Hamos spectrometer is increased by a factor of ∼15. This is a consequence of operation at a close to backscattering Bragg angle, where the solid angle per eV is larger. Moreover, the solid angle per eV is further enhanced for the von Hamos spectrometer because of the larger crystal size along the non-dispersive direction.

**Figure 3:**
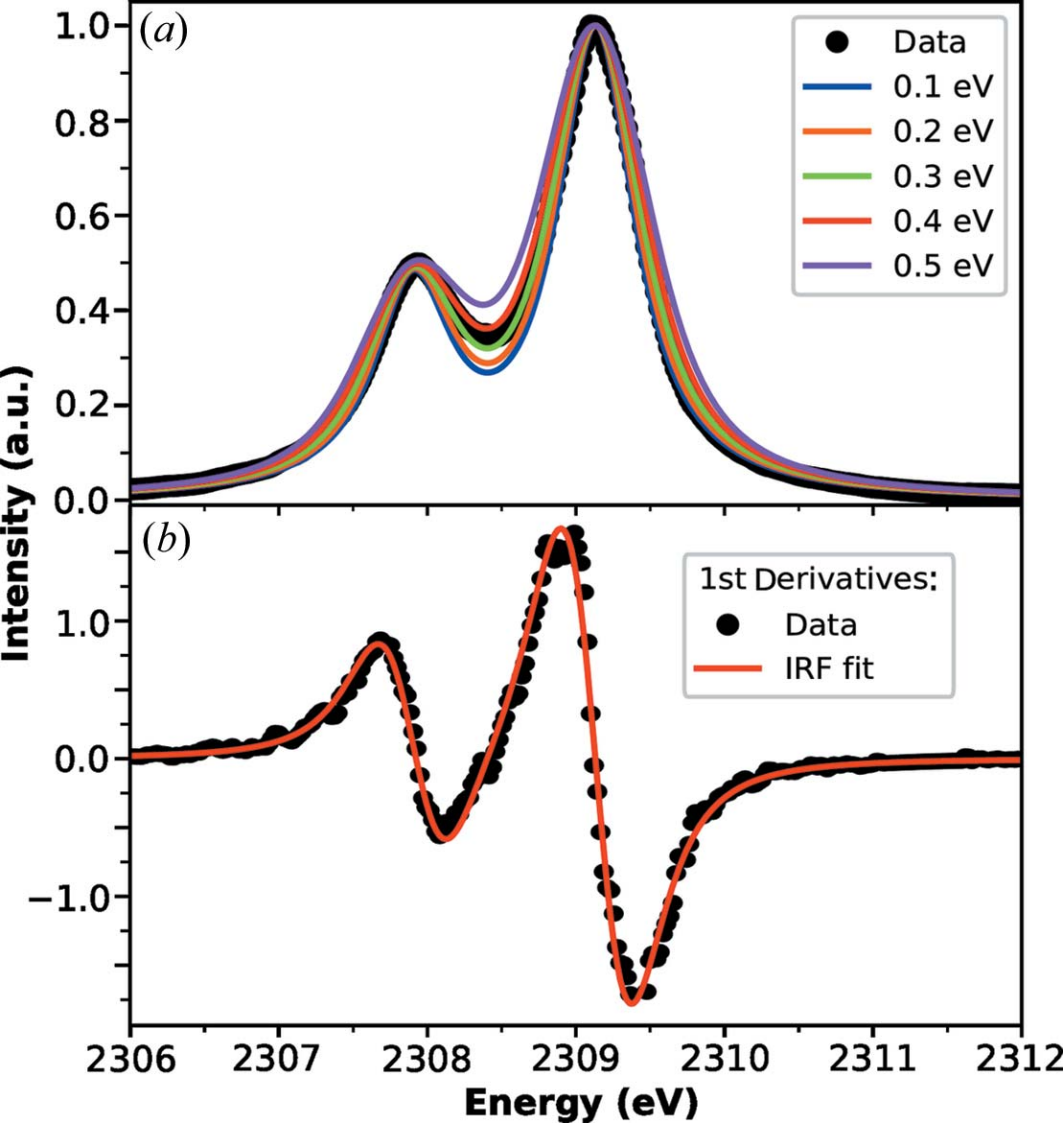
(*a*) Comparison of the measured sulfur *K*α spectrum using the lithium niobate von Hamos analyzer with spectra calculated by convolution of the natural Lorentzian lineshapes with Gaussian functions of varying widths to simulate the instrument response function. Best fit is obtained from a width of 0.29 eV. (*b*) Derivatives of the measured and best-fit simulated spectra show good agreement between their line shapes.

The resolving power of the instrument is confirmed by fitting the measured spectrum with simulated line broadening. Simulated spectra were produced through convolution of a Gaussian representing the instrument response function (IRF) with lifetime-broadened Lorentzian profiles (Campbell & Papp, 2001 ▸) at the *K*α_1_ and *K*α_2_ positions. Fig. 3 ▸ shows the effect of convolving increasingly broad IRFs with the natural linewidths in comparison with the collected (NH_4_)_2_SO_4_ XES. The best fit to the experimental data is obtained using a Gaussian IRF with 0.29 eV full width at half-maximum, producing an estimated resolving power of ∼8000 (*E*/Δ*E*). Close agreement between the derivatives of the simulated and measured spectra accentuates the similarity in their lineshapes.

**Figure 4:**
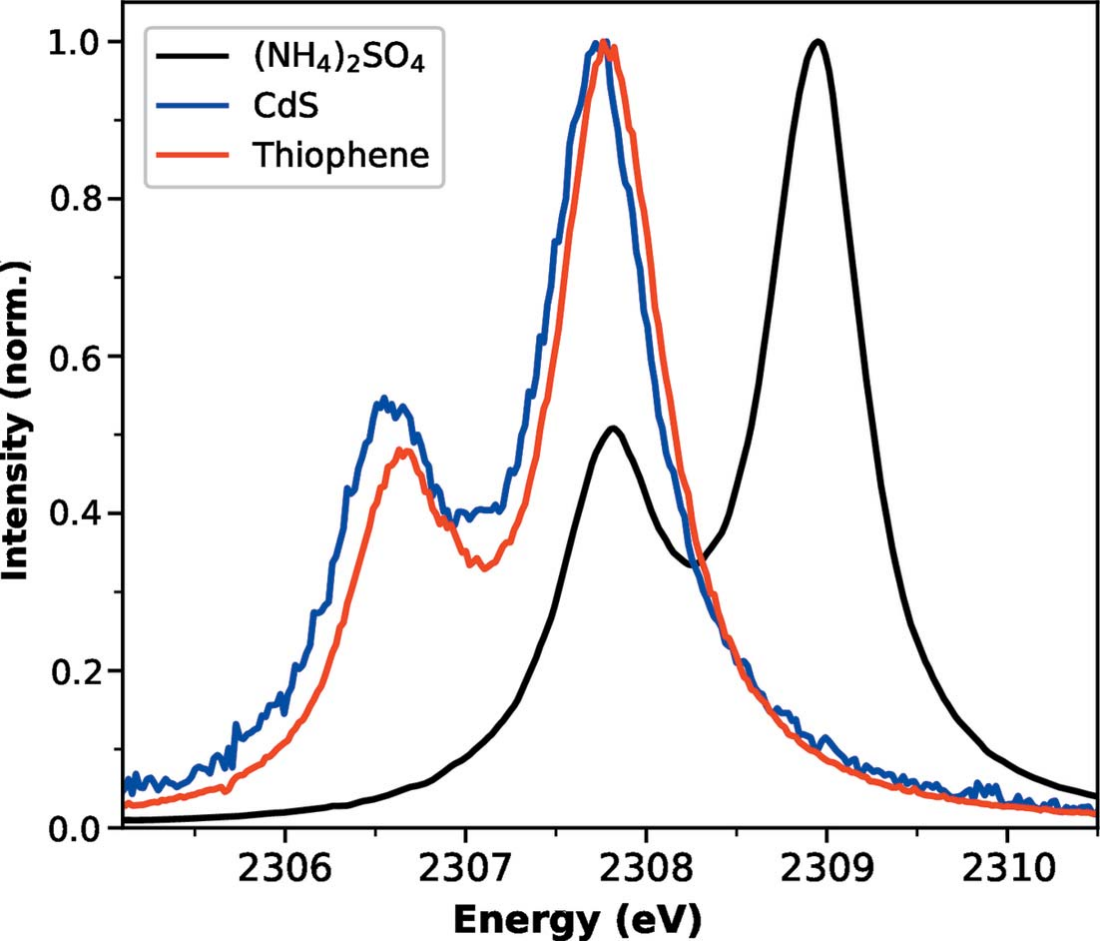
Normalized sulfur *K*α spectra of ammonium sulfate, CdS quantum dots and thiophene measured using the new spectrometer. The *K*α_1_ and *K*α_2_ peaks are fully resolved and are observed to shift in energy with the oxidation state of the resident sulfur atoms.

XES measurements were also performed on molecular and nanomaterial samples to demonstrate the versatility of the spectrometer. Thiophene was dissolved to 1*M* in toluene, while CdS quantum dots with an average diameter of 3.4 nm (Yu *et al.*, 2003 ▸) were concentrated at 80 m*M* in toluene. Signal was collected over ∼10 min for each sample. Both materials were flowed at 1 ml min^−1^ through a 50 µm liquid jet. Fig. 4 ▸ displays sulfur *K*α emission collected from the dissolved thiophene and CdS quantum dots by the von Hamos spectrometer, as well as the (NH_4_)_2_SO_4_. Sulfur *K*α_1_ and *K*α_2_ peaks are clearly resolved in each normalized spectrum. The *K*α peaks shift towards higher energies as the oxidation state of sulfur is increased. CdS and thiophene contain sulfur in a formal oxidation state of −2, although the aromaticity of thiophene delocalizes electron density from the sulfur atom to leave it slightly more positive (Zhang *et al.*, 2015 ▸). These emission spectra are accordingly ∼1.4 eV lower in energy than (NH_4_)_2_SO_4_, where sulfur atoms are in a +6 oxidation state (Alonso Mori *et al.*, 2009 ▸). A lower signal-to-noise ratio in the CdS spectrum is a result of the relatively low concentration. Moreover, at the time of the experiment, the epiX detectors were used in their very first stage of commissioning at LCLS and contributed substantial electronic noise to the signal.

## 4. Conclusions and outlook   

X-ray spectrometers operating in close to backscattering geometries across the entire tender X-ray energy range are greatly beneficial for studies that require high throughput and high energy resolution detection, such as time-resolved experiments and measurements on dilute systems. The spectrometer presented here, based on the use of a novel LiNbO_3_ 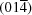 crystal analyzer, provides unprecedented efficiency for collecting XES and other photon-in photon-out signals in the tender X-ray regime. The finely tunable large *d*-spacing of LiNbO_3_ can enable the use of close to backscattering designs that provide enhanced sensitivity and resolution in an energy range that has been traditionally challenging to probe. Accordingly, the sulfur *K*α signal intensity collected with this instrument was increased by a factor of ∼15 as compared with an instrument based on a more traditional Johansson geometry.

The von Hamos spectrometer is particularly well suited for time-resolved photon-in photon-out measurements, as entire emission spectra can be recorded from individual X-ray pulses. The ability for rapid shot-to-shot acquisition of spectra with high efficiency in the tender X-ray region makes the instrument able to take advantage of the capabilities offered by next-generation lightsources such as LCLS-II and SwissFEL that provide ultrabright, ultrashort pulses at these energies. Furthermore, the tight line focusing of the von Hamos geometry permits the use of smaller area detectors when compared with other dispersive geometries, like Johansson, that require more extended detection areas. This can be advantageous for optimizing the signal-to-noise ratio, especially for the upcoming fast-readout cameras tailored for high-repetition-rate FEL applications. MHz pulse repetition rates at LCLS-II will facilitate photon-hungry measurements like RXES and HERFD-XAS, and the overall design of the spectrometer allows for experimental arrangements that easily couple these techniques with forward scattering measurements.

Based on the performance of the spectrometer described here, the design of a new advanced instrument to be deployed at LCLS-II, as well as at SSRL beamline 6-2 for steady-state and *operando* measurements, is underway. The planned capability upgrades include increasing the number of crystal analyzers and decreasing their radius of curvature to 250 mm. Such steps will increase the solid angle and further improve signal throughput by approximately another order of magnitude. Moreover, adopting a vacuum chamber enclosure instead of a He box will further improve attenuation issues. The upgraded instrument can therefore be expected to effectively measure samples down to millimolar concentrations. This instrument will be equipped with different lithium niobate cuts and other materials with appropriate *d*-spacings to provide tunability for measurements of other elements at near-backscattering Bragg angles throughout the tender X-ray regime.
